# Inflammatory Caspases Drive Pyroptosis in Acute Lung Injury

**DOI:** 10.3389/fphar.2021.631256

**Published:** 2021-02-05

**Authors:** Bohao Liu, Ruyuan He, Lin Zhang, Bo Hao, Wenyang Jiang, Wei Wang, Qing Geng

**Affiliations:** Department of Thoracic Surgery, Renmin Hospital of Wuhan University, Wuhan, China

**Keywords:** pyroptosis, acute lung injury, caspase, inflammasome, NLRP 3

## Abstract

Acute lung injury (ALI), a critical respiratory disorder that causes diffuse alveolar injury leads to high mortality rates with no effective treatment. ALI is characterized by varying degrees of ventilation/perfusion mismatch, severe hypoxemia, and poor pulmonary compliance. The diffuse injury to cells is one of most important pathological characteristics of ALI. Pyroptosis is a form of programmed cell death distinguished from apoptosis induced by inflammatory caspases, which can release inflammatory cytokines to clear cells infected by pathogens and promote monocytes to reassemble at the site of injury. And pyroptosis not only promotes inflammation in certain cell types, but also regulates many downstream pathways to perform different functions. There is increasing evidence that pyroptosis and its related inflammatory caspases play an important role in the development of acute lung injury. The main modes of activation of pyroptosis is not consistent among different types of cells in lung tissue. Meanwhile, inhibition of inflammasome, the key to initiating pyroptosis is currently the main way to treat acute lung injury. The review summarizes the relationship among inflammatory caspases, pyroptosis and acute lung injury and provides general directions and strategies to conduct further research.

## Introduction

Acute lung injury (ALI) is a life-threatening illness syndrome in intensive care units, which often is the main cause of acute respiratory distress syndrome (ARDS) (Butt et al., 2016). ALI/ARDS is characterized by severe inflammatory processes leading to diffuse alveolar injury, varying degrees of ventilation/perfusion divergence, poor pulmonary compliance and severe hypoxemia (Matthay et al., 2012). Pulmonary edema, increased alveolar permeability, leukocyte aggregation, epithelial injury and diffuse alveolar damage are typical pathological changes of ALI (Ware, 2006). There are many precipitating factors of ALI, such as severe shock, infection, mechanical injury, etc. (Kox et al., 2011; Kao et al., 2014) Over the years, a great number of articles have revealed that gram-negative bacterial infection is one of the most important factors leading to lung injury (Aujla et al., 2008). LPS, the major component of outer membranes of gram-negative bacteria, plays a key role in the process of inducing inflammatory response and leads to lung injury. That is why LPS has gradually become a major implement for inducing acute lung injury (Burgos et al., 2019) Lately, there has been improved interest in the relationship between the inflammatory response induced by LPS and pyroptosis (Zhao et al., 2018; Qiu et al., 2019; Muendlein et al., 2020). In consequence, elucidating the correlation between acute lung injury and pyroptosis may be helpful to find a more efficient way to treat acute lung injury from the etiology.

Caspases are a family of proteolytic enzymes that induce and transmit signals leading to cell death. They can be divided into apoptotic caspases (caspase-8, 9, 10, 3, 6, and 7) for inducing apoptosis and inflammatory caspases (caspase-1, 4, 5, and 11) for mediating pyroptosis (Ramirez and Salvesen, 2018). Caspase-1, formerly named interleukin-1b converting enzyme (ICE), initiates inflammation by affecting the maturation of pre-interleukins. The closest sequence identity is exhibited between caspase-1 and other members of inflammatory caspases including caspase-4, 5 and 11 (Black et al., 1989). Both caspase-1 mediating canonical pathway and caspase-4/11 or caspase-5 mediating non-canonical pathway can induce the occurrence of pyroptosis by using Gasdermin D (GSDMD) as the direct substrate (Man et al., 2017b). This review aims to elucidate the relationship among inflammatory caspases, pyroptosis and acute lung injury and to provide the latest research progress on the underlying mechanisms.

## The Function of Caspases in Pyroptosis

Pyroptosis is a form of programmed necrosis distinguished from apoptosis. Pyroptosis, which occurs in many types of cells in the body, is triggered for releasing inflammatory cytokines that clear cells infected by pathogen, prompting monocytes to reassemble at the site of injury (Ramirez and Salvesen, 2018). Pyroptotic cells are characterized by swollen cells with many vesicular protrusions, followed by the rupture of the cytoplasmic membrane and the flow of cytoplasmic content out of the cells. It is different from apoptosis in which cytoplasm membrane remains intact and apoptotic bodies are formed (Sangiuliano et al., 2014). Dependence on caspase-1 has long been an essential feature of pyroptosis (Bergsbaken et al., 2009). In recent years, the function of other inflammatory caspases and GSDMD, the key substrate and the direct executioner of pyroptosis, have been discovered to shed light on the mechanism of pyroptosis (Kayagaki et al., 2011; Baker et al., 2015; Pandeya et al., 2019; Xia, 2020).

### Caspase-1-dependent Pathway

Inflammatory caspases are referred to as the caspase-1 subfamily before. As mentioned above, caspase-1 was originally cloned for hunting an enzyme that acts on the proteolytic and mature of the pro-inflammatory cytokine IL-1β in human monocytes (Lamkanfi and Dixit, 2014). Different from some apoptotic caspases, caspase-1 contains a caspase recruitment domain (CARD) at the N-terminal end as well as a catalytic domain whose structure is very similar to that of the apoptotic promoter caspase-9 (McIlwain et al., 2013; Van Opdenbosch and Lamkanfi, 2019). The specific domain makes procaspase-1 form a dimer and be activated by pattern-recognition receptors (PRRs), when bacteria or viruses invade the body. Caspase-1 which is locked in the active form, directly cuts its substrate GSDMD (Shi et al., 2017). And the released N domain of GSDMD combines with phosphatidylinositol in plasma membrane to form membrane pore (Shi et al., 2017). The formation of membrane pores destroys the integrity of the cell membrane and the osmotic potential of the cell, so that water molecules enter the cell, causing swelling of the cell membrane, rupture of the cell membrane, and formation of balloon-like vesicles around the nucleus, accompanied by chromatin condensation and DNA fragmentation (Man et al., 2017a). Eventually the cells undergo osmotic lysis and the release of their contents leads to inflammatory cascades that are toxic to adjacent healthy cells and the entire organism (Wang et al., 2020; Xia, 2020). Meanwhile, caspase-1 cleaves the pro-inflammatory cytokines pro-IL-1β and pro-IL-18 that promotes the formation of mature IL-1β and IL-18, which can be released from pores with the cytoplasmic content (McIlwain et al., 2013; Winkler and Rösen-Wolff, 2015).

PRRs triggered by pathogen-associated molecular patterns (PAMPs) or danger-associated molecular patterns (DAMPs) is the starting point for activating caspase -1. PRRs can be divided into two categories according to the location of the subcell (Lamkanfi and Dixit, 2014). C-type lectin receptors (CLR) and Toll-like receptors (TLRs) present on cell membranes to detect extracellular stimuli, while the nucleotide-binding domain and leucine-rich repeat-containing (NLR) proteins, the AIM2-like receptor (ALR), and RIG-I-like receptor (RLR) are present inside the cell and stimulated by PAMPs or DAMPs (Lamkanfi and Dixit, 2014).

The canonical inflammasomes that participate in caspase-1-dependent pathway are principally composed of apoptosis-associated speck-like protein (ASCs), PAMPs and DAMPs receptors such as ALRs and NLRs, and procaspase-1 (Malik and Kanneganti, 2017). Inducing activation of caspase-1 is the most important function of the canonical inflammasome during pyroptosis. ASC containing a caspase-activation and recruitment domain (CARD) is recruited to form a polymer complex called “SPECK” by NLRs or ALRs which is considered as an indication of inflammasome assembly when a particular stimulus is detected (Kesavardhana and Kanneganti, 2017; Malik and Kanneganti, 2017). The common feature of NLR family members is that they contain a central nucleotide binding domain (NBD), whereas this family is further separated into NLR family pyrin domain-containing protein (NLRP) or NLR family CARD-containing protein (NLRC) receptors due to the differences in the N-terminal PYD domain (PYD) or CARD (Malik and Kanneganti, 2017). NLRP1, NLRP3 and NLRC4 have been widely studied to induce the formation of inflammasome and participate in the activation of caspase-1. Based on the lack of CARD structure, NLRP3 must rely on ASC with both pyrin and CARD as a bridge to recruit procaspase-1, while NLRP1 and NLRC4 are not (He et al., 2016b). The inflammasome directly involved in caspase-1 activation and pyroptosis has another name: pyroptosome. Unfortunately, the activation mechanisms and exact ligands of many inflammasomes are still not fully understood and are worth exploring.

### Caspase-1-independent Pathway

Caspase-1-independent pathway, named as non-canonical pyroptosis pathway, is induced by other members of inflammatory caspases including caspase-4, 5, and 11. Caspase-4/5/11, like caspase-1, structurally contains a CARD domain at the N-terminal end. Differently, human caspase-4 and 5 or their murine orthologs caspase-11 is directly stimulated by the cytoplasmic endotoxin of gram-negative bacteria, which directly lyses the substrate and leads to the occurrence of pyroptosis. Activation of non-canonical inflammasomes is required in caspase-1-independent pathway. The non-canonical inflammasome is assembled by direct interaction between procaspase-4/5/11 and the lipid A tail of LPS (Yi, 2017).

Caspase-11, which is specific to mice, is often used as a primary target for non-canonical pyroptosis pathway. Activated caspase-11 can not only induce the formation of cell membrane pores through the cleavage of GSDMD, but also promote K^+^ efflux via pannexin-1/ATP/P2X7 pathway, promote the maturation and release of IL-1β, and induce the reaction of intracellular inflammatory response (Oh et al., 2020). The N-terminal produced by cleavage from GSDMD has another function for promoting the proteolytic activation of caspase-1 (Wang et al., 1998; Kayagaki et al., 2011). Thus, caspase-11 is also regarded as an upstream regulator of caspase-1 activation (Shalini et al., 2015).

Caspase-4 and caspase-5 play an important role in the caspase-1-independent pyroptosis pathway in humans. Studies have shown that caspase-4 is directly involved in the detection of LPS, while caspase-5 has no detectable role in identifying these pathogens which can be specifically regulated by IFN–γ and LPS (Lin et al., 2000; Matikainen et al., 2020). Caspase-4 promotes its own activation through direct interaction with LPS and exhibits a higher affinity with tetra-acylated LPS, showing a wider range of specificity than caspase-11 (Matikainen et al., 2020; Oh et al., 2020). However, many mechanisms of caspase-4/5/11 *in vitro* and *in vivo* remain unclear.

### Other Pathway

It has been reported that caspase-3, as a key regulator of apoptosis, can also be involved in the mediation of pyroptosis. As we all know, caspase-3 is at the end of caspase cascades during the regulation of apoptosis and can be cleaved and activated by caspase-9, while caspase-3 appears as an upstream regulator in pyroptosis (Rogers et al., 2017; Frank and Vince, 2019). After induction with TNF-α or chemotherapeutic agents, GSDME, initially named as DFNA5 (Deafness Autosomal Dominant 5), is directly cleaved by caspase-3 specifically to produce the GSDME-N fragment (Wang et al., 2017a; Rogers et al., 2017). The fragment performs the same function as the activated GSDMD, penetrates the cell membrane and mediates pyroptosis (Wang et al., 2017a). Recent studies have shown that ATP induces caspase-3-induced pyroptosis after NLRP3 is blocked (Wang et al., 2017a). This pathway therefore seems to be understood as an alternative mechanism for pyroptosis. The exact mechanism of this pathway remains largely unknown.

## The Role of Inflammatory Caspases and Pyroptosis in ALI

Inflammatory caspases have been proved to produce a marked effect in inducing inflammatory response in cardiovascular disease, brain injury, liver injury and kidney injury (Irrera et al., 2020; Shojaie et al., 2020). And inflammasomes are non-substitutable in the process of pyroptosis caused by inflammatory caspases in acute lung injury. Moreover, a lot of research revealed that the inducing factors and manifestations of pyroptosis are slightly dissimilar in different types of cells, including macrophages, neutrophils, and endothelial cells.

### Pyroptosis in Macrophages

**FIGURE 1:**
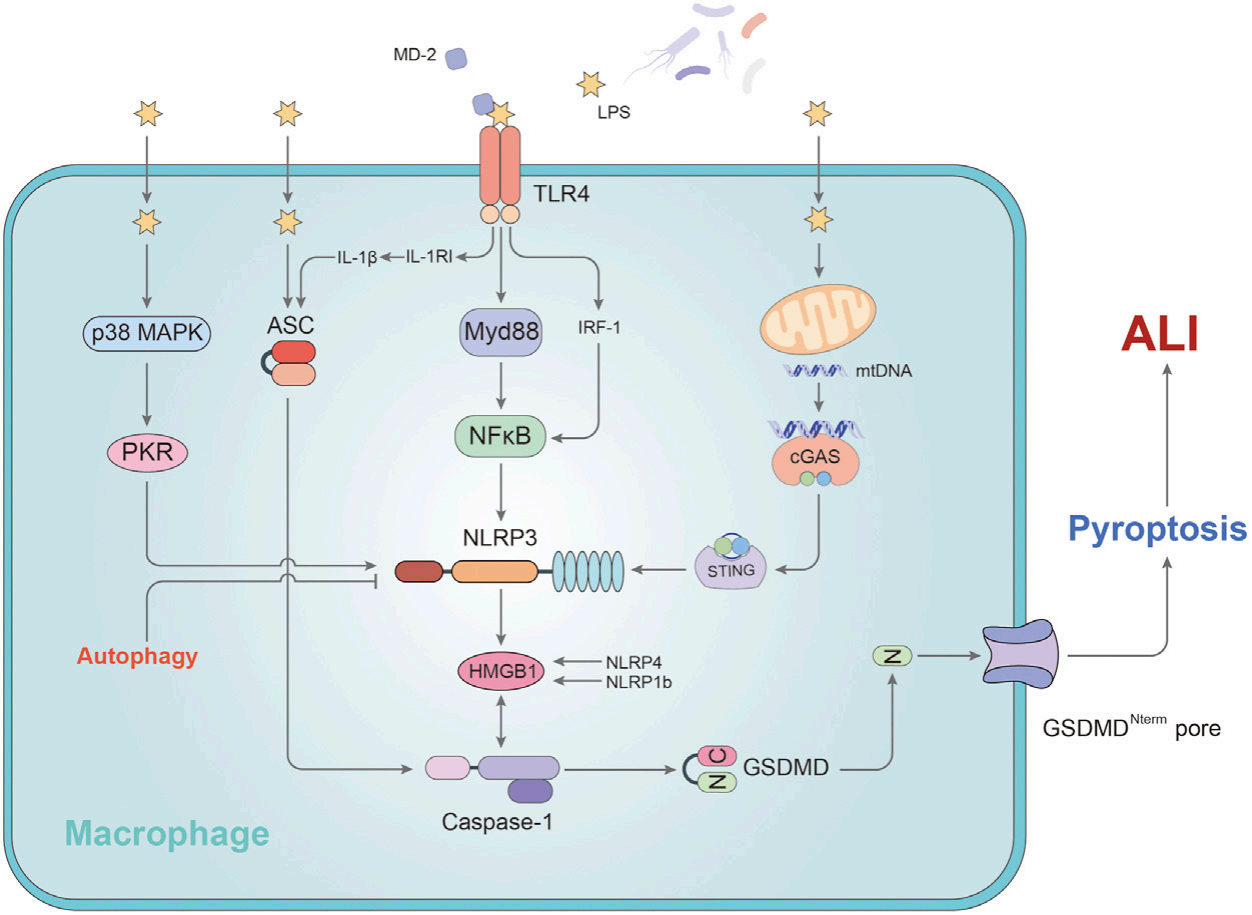
The mechanism of pyroptosis affecting alveolar macrophages in ALI. In AMs, LPS promotes the activation of caspase-1 by regulating MD2, ASC, and NLRP3, leading to pyroptosis. P38/MAPK and TLR4-Myd88 signaling pathway participate in stimulating the development of pyroptosis via NLRP3. NLRP3, an important regulatory target, can be activated by mtDNA or cleared via autophagy pathway. HMGB1 plays a key role in this process. Stimulation of HMGB1 secretion is dependent on NLRP4/NLRP1b rather than ASC. IL-1RI regulated by TLR4 sensitizes AMs to IL-1β and facilitates to the formation of ASC pyroptosome causing the exaggeration of AMs pyroptosis. GSDMD-N fragment, derived from caspase -1’s cleavage of GSDMD, is transported to the cell membrane and initiates pyroptosis in AMs.

Macrophages are essential for natural immunity and host defense and play an important role in the lungs and the alveolar cavity. The differentiation, phenotype, function and cell-cell interaction of macrophages are indispensable in the initiation and resolution of pulmonary inflammation (Aggarwal et al., 2014). A growing body of research suggests that alveolar macrophages (AMs) have a profound impact on the development and prognosis of acute lung injury by releasing inflammatory cytokines and regulating other immune cells (Tang et al., 2017; Hung et al., 2018; Kojima et al., 2018) (Figure 1).

After activation of the inflammatory corpuscular pathway in pyroptosis, two lyzed subunits called p10 and p20 originate from processed procaspase-1, which is the main inducement factor of the release of IL-1β and IL-18 into the extracellular environment (Miao et al., 2011). A study has shown that p10 in the AMs activated by caspase-1 of the mice successfully induced ALI/ARDS was markedly increased after being processed by LPS, while the caspase-1 inhibitor Ac-YVAD-CMK could restrain this phenomenon (Wu et al., 2015). The authors found that NLRP3 and ASC were highly expressed following LPS through Western Blotting and immunofluorescence. In conclusion, alveolar macrophage pyroptosis may be involved in the development of acute lung injury and NLRP3/ASC inflammasomes participate in the process (Wu et al., 2015).

Moreover, the study of Lei Hou et al. indicated that NLRP3/ASC-mediated AM pyroptosis induced High-mobility group box 1 (HMGB1) secretion from AM in ALI (Hou et al., 2018). HMGB1 as one of DAMPs can promote the activation of NLRP3 and the activation of caspase-1, thus feedback positive to augment AM pyroptosis, aggravate the inflammatory response and cause the worsening of ALI (Qu et al., 2019). The study showed that knockdown of ASC or inhibiting the function of NLRP3 inflammasome can mitigate the AM pyroptosis and reduce HMGB1 release, leading to decrease the inflammation in the ALI (Hou et al., 2018). However, the secretion of HMGB1 from macrophage is seemingly independent of ASC, but dependent on NLRP4 or NLRP1b and caspase-1 (Lamkanfi et al., 2010; Van Opdenbosch et al., 2014). In addition, a study from Dandan Li et al. showed that p38-MAPK inhibitor SB203580 reverses the LPS-induced expression of caspase-1 and NLRP3 in AM. The blockage of p38-MAPK signaling pathway can reduce the expression level of pro-inflammatory cytokines and the assembling of NLRP3 inflammasomes from the lung tissues of inflammatory mice (Li et al., 2018). The authors concluded that p38-MAPK may participate in the promotion of ALI and lung inflammation through regulation of the NLRP3 inflammasome and AM pyroptosis. Double-stranded RNA-dependent kinase (PKR) has been shown in another study to be an effective target for blocking this pathway in alveolar macrophages (Zeng et al., 2019).

Interleukin-1β (IL-1β) is a pro-inflammatory mediator of inflammation which is released extracellular after pyroptosis occurs (Bergsbaken et al., 2009). The activation of IL-1β is closely related to the proteolysis of caspase-1 and the assembly of the inflammasomes which arises from the cutting of caspase-1 at amino acid position 116 (Delaleu and Bickel, 2004; Guo et al., 2015). It plays a main role in stimulating white blood cells to express various cytokines and chemokines and promoting the immigration of white blood cells to organs, thus exaggerating the development of inflammation (Delaleu and Bickel, 2004). A study investigated the influence of LPS-induced IL-1β release on the development of AM pyroptosis and lung inflammation (He et al., 2016a). The study showed that Toll-like receptor 4 (TLR4) up-regulates the release or expression of IL-1β and IL-1RI on the AM surface. IL-1RI upregulation sensitizes AMs to IL-1β and facilitates to the formation of ASC pyroptosome causing the exaggeration of AMs pyroptosis, which subsequently exacerbates ALI (He et al., 2016a). Xingying He et al. demonstrated a new mechanism of endotoxin induced innate immunity that a correlation between the secondary upregulation of IL-1β/IL-1RI signals and AM pyroptosis in lung injury through TLR4-MyD88-NF-ĸB dependent signaling (He et al., 2016a). According to their results, MyD88 is related to mediate the process of TLR4-IL-1RI cross-talk and NF-κB involves in facilitating the upregulation of IL-1RI induced by TLR4. Additionally, another article has concluded that myeloid differentiation protein 2 (MD-2), one of the core factors required for TLR4 to recognize LPS, heightens caspase-1, NF-κB p65 protein, NLRP3 and MyD88 expression and helps regulate the activation of LPS-induced NLRP3 inflammasome and the inflammatory response in AMs via MyD88/NF-κB signaling pathway (Luo et al., 2017). And this outcome eventually led to the secretion of IL-1β and pyroptosis of AMs. Coincidentally, another study also indicated that interferon regulatory factor-1 (IRF-1) plays an important role in mediating alveolar macrophage pyroptosis during acute lung injury (Wu et al., 2016). As a gene known to participate in the interferon (IFN) pathway, IRF-1 straightly adjusts alveolar macrophage pyroptosis and caspase-1 activity structurally by IRF-1-binding element (IRE) from casapse-1 gene. And the study identified that TLR4 is essential in the activation of IRF-1. MyD88 has long been proven to recruit the IRF-1 transcription factors to activate the TLR4 pathway (Harroch et al., 1995; Szekely et al., 2018). Therefore, IRF-1 may be a hub gene between LPS-TLR4 signaling and caspase-1-dependent pyroptosis pathway in alveolar macrophages.

As a highly conserved metabolic degradation system, autophagy has been shown in recent years to maintain homeostasis and protect cells under abnormal stimulation by a large amount of evidence (Duan et al., 2020). Wei Peng et al. found autophagy of alveolar macrophages played a protective role as the upstream regulator of pyroptosis in acute lung injury induced by mitochondrial damage-associated molecular patterns (MTDs) (Peng et al., 2020a). Enhanced autophagy in AMs inhibits the function of the NLRP3 inflammasome by degrading and clearing the activated NLRP3. And from that, caspase-1 and IL-1β precursors cannot be cleaved into the active form, thus inhibiting the caspase-1-mediated pyroptosis pathway (Peng et al., 2020a). It is expected that inhibition of pyroptosis by activating one of the upstream pathways of autophagy may become a new strategy for the treatment of ALI.

Our team previously illustrated that a new mechanism in macrophages to explain how mitochondrial DNA (mtDNA) activates the pyroptosis pathway to aggravate the inflammatory response of ALI (Ning et al., 2020). In our study, LPS stimulation causes mtDNA in mitochondria to leak into the cytoplasm and be identified by the sensory receptor of cytosolic DNA (cyclic GMP-AMP synthase, cGAS) (Ning et al., 2020). Stimulator of interferon gene (STING) is triggered by cGAS and its phosphorylation levels rise (Jena et al., 2020; Ning et al., 2020). LPS also upregulated the expression of transcription factor c-Myc, thus directly promoting the expression level of STING. The upregulation and activation of STING activates NLRP3 inflammasome and caspase-1, eventually leading to cell pyroptosis and ALI (Jena et al., 2020; Ning et al., 2020). Based on the CGAS-STING axis, our team will further explore the upstream mechanism of macrophage pyroptosis in acute lung injury.

In summary, the main function of macrophages in lung tissue is to preserve homeostasis and activate autoimmunity under pathogen stimulation. The suppression of inflammatory response in macrophages is crucial for the treatment of acute lung injury. There is still high research value in the field of ALI on the mechanism of inflammatory response pathway and AM.

### Pyroptosis in Endothelial Cells

**FIGURE 2:**
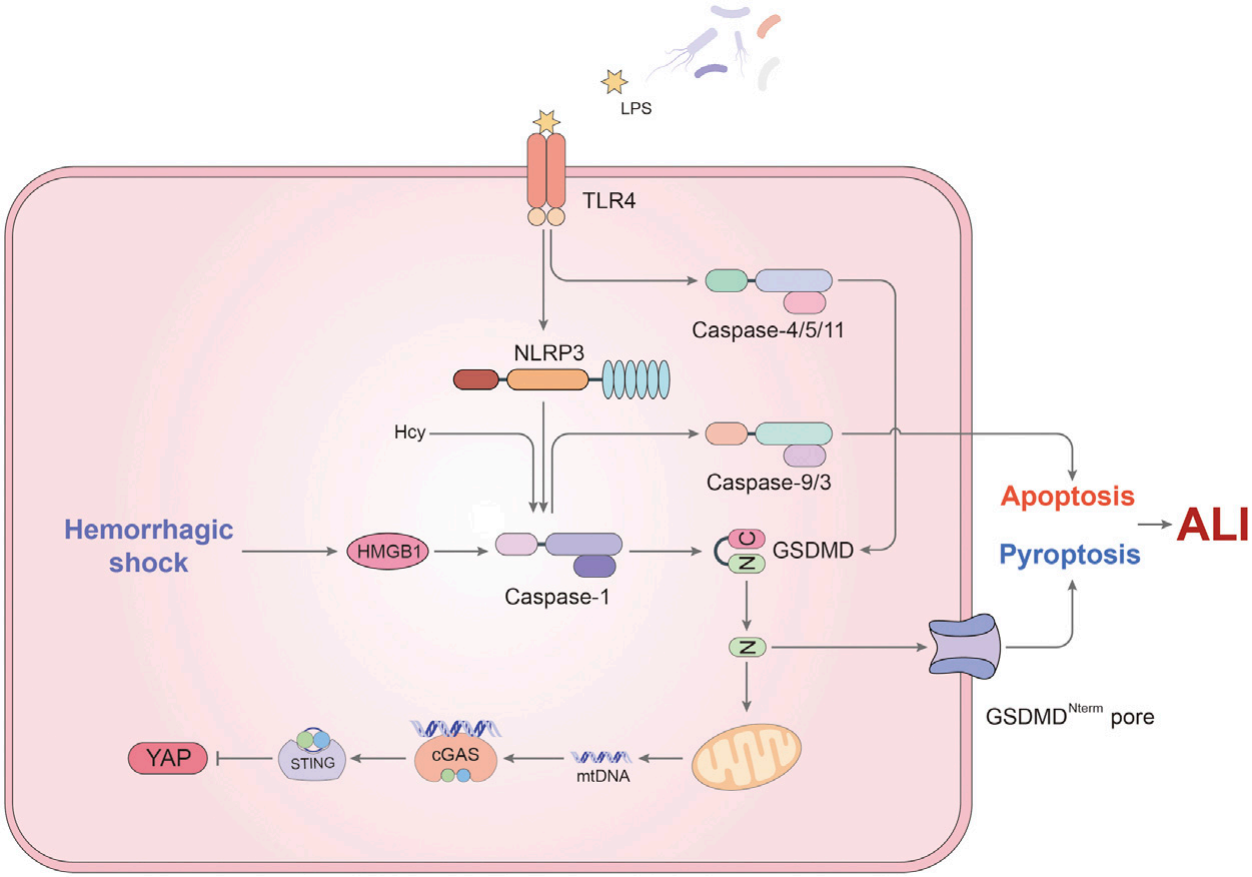
The mechanism of pyroptosis affecting endothelial cells in ALI. In ECs, caspase-1 not only induces pyroptosis, but also participates in the regulation of apoptosis. LPS and hemorrhagic shock (HS) are two major triggers for activation of caspase-1. TLR4 on the cell membrane recognizes LPS and up-regulates NLRP3 expression, thus inducing caspase-1-mediated pyroptosis. Besides, HS promotes the formation of inflammasomes via HMGB1/RAGE signaling pathway. Cleaved caspase-1 is responsible for the downstream caspase-9 and 3 activation leading to apoptosis in EC when Hcy and LPS stimulate simultaneously. Meanwhile, non-canonical pyroptosis pathway mediated by caspase-4/5/11 also occupies a certain role in endothelial pyroptosis. GSDMD cleaved by caspase-11 has another function that promotes mtDNA releases. Through cGAS/STING axis, mtDNA inhibits the expression of YAP, resulting in endothelial cell proliferation disorders. In addition to the above, the effect of pyroptosis on the pulmonary endothelial barrier still needs to be studied.

A single layer of fused endothelial cells (EC) covered the inner wall of pulmonary microvessels, called pulmonary endothelial barrier. ECs are responsible for the mediation of vascular tone, permeability, ventilation–perfusion matching and interactions with blood-borne cells (Maniatis and Orfanos, 2008; Muller-Redetzky et al., 2014). Pulmonary ECs account for about 50% of all lung cells, which have the function of accept total cardiac output. As a result, lung endothelial cells are most likely to be disturbed by bacterial endotoxins and circulating pathogens. Pulmonary endothelial barrier dysfunction caused by extensive ECs death is a hallmark of acute lung injury (Liu et al., 2015). Considering the induction of pyroptosis by LPS, a number of publications put attention on the impact of inflammatory caspases on ECs in ALI (Figure 2).

A study from Jie Yang et al. sheds light on the important role of hemorrhagic shock (HS) and EC pyroptosis in ALI (Yang et al., 2016). HS tends to make the patient more vulnerable to lung damage because an exaggerated response is initiated to respond a second infectious stimulus (Rubenfeld et al., 2005). In the study, the authors documented that the activation of caspase-1 in ECs is due to two sources. On the one hand, hemorrhagic shock promoted the secretion of HMGB1 which forms pyroptosome and activates caspase-1 through RAGE signaling and originated the endocytosis of HMGB1 by endothelial cells (Yang et al., 2016). On the other hand, caspase-1 activation was induced by LPS via TLR4-NLRP3 signaling pathway in ECs (Yang et al., 2016). More importantly, this article highlights the activation of caspase-1 in endothelial pyroptosis. Through the measurement of BALF protein concentration, lung tissue myeloperoxidase (MPO) activity and lung wet/dry ratio, they concluded that EC pyroptosis induced by HS could lead to acute lung injury (Yang et al., 2016).

Caspase-1 simultaneously plays different roles in the destruction of lung endothelial cells, not only causing pyroptosis. In a previous study, caspase-1 was related to induce ECs apoptosis and the release of IL-1β/IL-18 in lung ECs (King et al., 2003). This process seems to be connected with homocysteine (Hcy) (Xi et al., 2016). It has been reported that Hcy promotes the apoptosis of ECs and inhibits ECs growth leading to endothelial dysfunction (Tan et al., 2006; Cheng et al., 2015). In addition, Hcy can also induce inflammasome assembly and caspase-1 activation, which is synergistic with LPS. Cause it is the earliest event with higher hierarchy in caspase cascade in EC, caspase-1 activation may be responsible for mediating Hcy/LPS- induced pyroptosis/apoptosis and the downstream caspase-9 and -3 activation in EC (Xi et al., 2016). What was novel about this study was that the authors named the kind of programmed cell death which was mediated by caspase-1 through increased ROS as pyrop-apoptosis. The result also provided evidence for crosstalk between inflammatory and apoptotic caspases. It is worth mentioning that IL-33 can subdue the function of caspase-1 in ECs, which mediates Group 2 innate lymphoid cells (ILC2) expansion in the lungs acting through its receptor ST2 (Lai et al., 2018). The increase of ILC2 in the lung contribute to secrete IL-9, which inhibits the activation of caspase-1 to prevent pulmonary ECs from the occurrence of pyroptosis or apoptosis (Lai et al., 2018).

After pyroptosis occurs, the downstream pathway of caspase-1 also plays a pro-inflammatory role. Activated caspase-1 in ECs leads IL-1β to be secreted and performs its physiological functions. After binding with its specific receptor, IL-1β up-regulates the expression of adhesion factors in pulmonary capillary endothelial cells, thus promoting chemotaxis and activation of inflammatory cells into lung tissues, releasing a large number of inflammatory factors and triggering the inflammatory cascade amplification effect (Zhu et al., 2012). At the same time, IL-1β can also destroy the cell surface localization of vascular endothelial (VE)- cadherin by promoting endocytosis, and increase capillary permeability (Zhu et al., 2012).

Microparticles/microvesicles (MPs/MVs) are minor membrane-covered structures secreted from activated or dead cells (Distler et al., 2006). Microparticulate caspase-1 is a novel effective form for mononuclear macrophages to transmit death signals and caspase-1 to mediate cell death (Sarkar et al., 2009). Caspase-1 packaged into circulating microparticle is almost released from mononuclear phagocyte. The mechanism of microparticulate caspase-1 acting on lung ECs has been reported in recent years (Mitra et al., 2015; Mitra et al., 2018). Stimulated monocytic cells release activated caspase-1, GSDMD, ASC and other inflammasome proteins in target specific microparticles. This kind of exogenous caspase-1 induces endothelial cells apoptosis and pyroptosis. ASC, the inflammasome adaptor protein, and the capsulation of caspase-1 are vital for apoptotic and uptake function (Mitra et al., 2015). Once the microvesicle structure is destroyed, the novel caspase-1-dependent apoptosis is completely inhibited. In the process of microparticulate caspase-1 mediating pyroptosis of endothelial cells, cleaved GSDMD is categorically necessary (Mitra et al., 2018). As we all know, GSDMD is related to the formation of plasma membrane pores. Therefore, the authors speculated that the pore forming capacity of GSDMD is significant to the aptitude of MPs to locate the target (Mitra et al., 2018). In summary, the activation of caspase-1 regulates GSDMD cleavage, while cleaved GSDMD regulates the release of MPs encapsulating caspase-1 (Mitra et al., 2018). However, it would be further studied to confirm how does GSDMD act on MPs and whether it produces pores.

As more and more studies have been published on caspase-1-independent pathway of pyroptosis, the effect of caspase-4/5/11 in ECs draws attention recently. It was found that LPS could up-regulate the expression of caspase-11 in mouse endothelial cells via TLR4 and then induce pyroptosis of endothelial cells. In this process, caspase-11 is indispensable, nevertheless TLR4 can be substituted by TLR3 ligand poly and TLR2 ligand Pam3CSK4 (Cheng et al., 2017). Caspase-11 plays the central part in the mechanism of EC pyroptosis induced by endotoxin which is the causation of lung vascular hyperpermeability and mortality. Meanwhile, the release of MPs from pyroptotic ECs is significantly increased under the action of caspase-11.

As mentioned in the previous section, mtDNA still promotes inflammation and damage in the caspase-11-mediated pyroptosis pathway of endothelial cells. After LPS stimulation, caspase-11 is activated and participates in the assembly of atypical the non-canonical inflammasome (Huang et al., 2020). The resulting GSDMD-NT fragment not only induces the formation of plasma membrane pores, but also locates to mitochondria and induces mtDNA release into the cytoplasm (Huang et al., 2020). MtDNA, on the one hand, further promotes the process of cell pyroptosis, and on the other hand inhibits the expression of YAP through the cGAS-STING signaling pathway (Huang et al., 2020). The suppression of YAP signaling, a known pathway that regulates cell proliferation (LaCanna et al., 2019), reduces vascular repair and endothelial cell proliferation. To sum up, caspase-11 plays an important role in the anti-regeneration and pyroptosis of endothelial cells.

### Pyroptosis in Neutrophil

Increasing evidence has shown that neutrophils are increasingly thought to be complex, multifaceted cells that play an important role in initiating and sustaining inflammation and promoting regression, though paradoxically (Vassallo et al., 2019). In lung injury, neutrophils not only cause lung tissue damage by mediating the inflammatory response (Summers et al., 2014), but also eliminate inflammation by recruiting monocytes and releasing a cluster of “specialized pro-resolving mediators” (SPMs) (Dalli and Serhan, 2012). As early as in 2002, the study of Sarah J. Rowe et al. found that up-regulation of the mRNA for IL-1β is significant after LPS stimulation in caspase-1-deficient mice, but their neutrophils released IL-1β minimally (Rowe et al., 2002). The authors thought the result may be related to the mice's resistance to intraperitoneal injection of endotoxin shock. However, caused by the discovery of the concept of pyroptosis, the minimum release of IL-1β from their neutrophils probably resulted on that the defect of caspase-1 inhibited the canonical pathway of pyroptosis. This is the first time that inflammatory caspases and pulmonary neutrophils have been associated with lung injury.

Recently, neutrophils was reported to achieve host protection facilitated by NLRC4/caspase-1-dependent IL-β secretion (Chen et al., 2014). In the acute lung infection induced by *P. aeruginosa* PAO1, neutrophils, rather than AMs, are indispensable for host protection. And neutrophil pyroptosis in the caspase-1-dependent manner is attributed to PAO1 flagellin for activating caspase-1 inflammasome and TLR5 which regulates PAO1 uptake under NOX2-deficient conditions (Ryu et al., 2017). The secretion of IL-1β/IL-18 increased significantly through NLRC4-caspase-1 pathway and NLRP3-caspase-11 pathway while neutrophil pyroptosis happens. It was concluded that PAO1-induced neutrophil pyroptosis may be activated by the collaborative recognition of PAO1 flagellin through TLR5 and NLRC4 (Ryu et al., 2017).

On the other hand, neutrophils, as important effector cells in immune response, recruit and exert immune function at the injured site in the process of acute lung injury (Williams and Chambers, 2014). The chemokine CXCL12 is involved in the regulation of neutrophil migration, as is its receptor CXCR4 (Tulotta et al., 2019). NLRP3-mediated pyroptosis has been shown in studies to promote the expression of CXCL12 in neutrophils, thereby inducing neutrophil migration and aggravating the severity of acute lung injury(Peng et al., 2020b). However, in the model of lung ischemia–reperfusion (IR), the recruitment of neutrophils induced by pyroptosis is a very effective defense mechanism against bacterial infections after injury (Tian et al., 2017). Minimal lung damage may follow if the post-IR pulmonary environment is sterilized (Tian et al., 2017). Otherwise dormant neutrophils would be transformed into activated neutrophils in the presence of a pathogenic toxin, which may help protect against infection (Tian et al., 2017).

## The Treatment of ALI by Inhibiting Inflammasomes

As mentioned above, inflammasomes are indispensable in the process of inflammatory caspases-induced pyroptosis leading to lung injury. More recently, there has been a growing number of studies focusing on inhibition of the inflammasomes or blockage of signaling pathways to find out new treatment strategies for ALI (Samanta et al., 2018; Zhang et al., 2018; Gao et al., 2019; Wang et al., 2019; Yao and Sun, 2019; Niu et al., 2020; Shao et al., 2020).

### NLRP3 Inflammasome

NLRP3 inflammasome is the most studied inflammasome, which activates caspase-1 in the caspase-1-dependent pyroptosis pathway. The activation of NLRP3 inflammasomes requires many molecular or cellular events as triggers, such as K^+^ outflow, Ca^2+^ signals, reactive oxygen species stimulation or lysosome rupture, etc. (He et al., 2016b) In addition, there is the non-canonical NLRP3 inflammasome that can be triggered by caspase-11 without the regulation by TLR4 (Kayagaki et al., 2013). The inhibition of NLRP3 inflammasome is currently attracting considerable interest as an effective treatment for acute lung injury.

#### NLRP3 in LPS-Induced ALI

LPS-induced ALI is the most common and has the most pronounced inflammatory response in all kinds of ALI. Yu-chang Wang et al. found that dihydromyricetin (DHM) could reduce the protein expression of caspase-1, IL-18, IL-1β, NLRP3, ASC and pro-GSDMD to inhibit the inflammation in ALI and the inhibiting action was grounded on the defeat of NLRP3 inflammasome-dependent pyroptosis signaling pathway (Wang et al., 2019). Glycoprotein extracted from Chinese Yam, a kind of Chinese medicine homologous food, can also attenuate the secretion of NLRP3 in ALI and the activation of LPS-induced NF-κB/TLR4 signaling pathway can be restrained (Niu et al., 2020). Both DHM and glycoproteins are concerned because of their immunomodulatory effects, and have a negative effect on MPO activity in tissues. With the increasing research on inflammasome and lung injury, numbers of anti-inflammatory molecules including melatonin, oridonin or apocynin have been reported to suppress the NLRP3 inflammasome activation to achieve the purpose of attenuating acute lung injury (Zhang et al., 2016; Jin et al., 2019; Yang et al., 2019).

#### NLRP3 in Ventilator-Induced Lung Injury

Although mechanical ventilation (MV) is an effective treatment for acute lung injury at present, it will lead to the recruitment of inflammatory cells and induce the release of inflammatory mediators and cytokines, thus leading to the occurrence of acute lung injury when tidal volume is high (Liu et al., 2019). NLRP3, as an upstream regulator of caspase-1-meidated pyroptosis pathway, is an important target for reducing ventilator-induced lung injury (VILI) (Kuipers et al., 2012). Mechanical stretching induced by the ventilator induces the release of reactive oxygen species in the mitochondria of the cell (Wu et al., 2013). Mitochondrial activation and extracellular aggregation of IL-1β are highly dependent on the released mtROS (Wu et al., 2013). With one accord, a recent study suggests that oxidative stress exacerbates VILI via NLRP3 pyroptosis pathway (An et al., 2019). Therefore, inhibiting the accumulation of ROS or the occurrence of oxidative stress would be an effective way to inhibit the function of NLRP3. Moreover, salidroside and dopamine have also been reported to play a protective role in VILI by inhibiting the expression of NLRP3 and reducing the release of inflammatory factors (Wang et al., 2017b; Yang et al., 2017).

#### NLRP3 in Hyperoxia-Induced Lung Injury

In the hyperoxia-induced lung injury (HALI) model, NLRP3 has been shown to induce HALI by regulating chemotaxis and recruitment of immune cells and IL-1β expression levels (Fukumoto et al., 2013). P2X7, a kind of membrane receptor, is responsible for the activation of NLRP3 after exposure to hyperoxia (Galam et al., 2016). Caffeine and lycium barbarum polysaccharides (LBP) can protect hyperoxia-induced lung tissue from oxidative injury by inhibiting NLRP3 inflammasome (Hong et al., 2019; Chen et al., 2020). Interestingly, another study has shown that activated NLRP3, in addition to activating caspase-1, also plays a protective role in HALI by activating the Stats pathway in lung epithelial cells (Mizushina et al., 2015). The lack of NLRP3 greatly decreases the expression of chemokines in macrophages and neutrophils, thereby inhibiting Stat3 activation, leading to a weakening of HALI's protective mechanism (Mizushina et al., 2015). As a result, NLRP3^−/−^ mice show less lung inflammation and inflammatory cell infiltration, but a higher fatality rate (Mizushina et al., 2015).

### Other Inflammasome

Compared with the NLRP3 inflammasome, much less research has been done on inhibiting other inflammasomes that cause pyroptosis. A publication from Jian Gao et al. identified that AIM2 inflammasome could be inhibited by andrographolide to achieve the amelioration of acute lung injury (Gao et al., 2019). Cause for the function of preventing AIM2 from migrating to the nucleus, andrographolide significantly inhibited AIM2 inflammasome activation and lung macrophage pyroptosis (Gao et al., 2019). This is a representative study on AIM2 inflammasome for the treatment of acute lung injury in recent years. NALP3 inflammasome has the function of activating pathological processes where excessive IL-1β is produced (Campbell et al., 2016). Recently, a study from Leifang Zhang et al. revealed that suppressor of cytokine signaling-1 (SOCS-1) limits the assembly of NALP3 inflammasomes and significantly inhibits the activation of caspase-1 in smoke inhalation-induced acute lung injury as a negative regulator of pro-inflammatory cytokine signal transduction (Zhang et al., 2018). Beyond that, further research is needed on the treatment and improvement of acute lung injury by inhibiting inflammasome-induced cell death.

## Discussion

ALI is characterized by extensive inflammatory cell infiltration in lung tissue and damage to alveoli and endothelium resulting from an exaggerated inflammatory reaction. In the absence of a corresponding inflammatory response, apoptosis alone is undoubtedly inappropriate to describe the underlying mechanism of ALI. Pyroptosis induced by inflammatory caspases is responsible for swelling of cells and release of pro-inflammatory contents compared with apoptosis. With the increase of research on GSDMD, the process and function of pyroptosis is becoming much clearer.

Recently, a study showed that caspase-8 also mediates LPS-induced pyroptosis of macrophages, and this process does not require the involvement of other apoptotic caspases (Muendlein et al., 2020). Although the exact process and whether it occurs only in macrophages, remain unclear, the result undeniably shattered the previous understanding of pyroptosis and apoptosis. The crosstalk between inflammatory caspases and apoptotic caspases has attracted the interest of many scholars.

This review focuses on recent advances in the study of pyroptosis in ALI and the treatment via inflammasome. Although these recent advances provide evidence for the role of inflammatory caspases and pyroptosis in acute lung injury, many details remain indefinite. Most of the current studies have focused on caspase-1-dependent signaling pathways, while the non-canonical pathway induced by caspase-11/4/5 is rarely reported. Furthermore, the selection mechanism of canonical or non-canonical pyroptosis pathways in lung tissue and the role of pulmonary epithelial pyroptosis in ALI is still awaiting further study.

At present, COVID-19 is causing serious outbreaks around the globe, affecting people’s health and development in many countries. Whether the resulting ALI/ARDS can be improved by blocking the pyroptosis signaling pathway deserves immediate experimental verification (López-Reyes et al., 2020). Therefore, further elucidation of the role of pyroptosis in lung injury is necessary in order to better understand its mechanism and how to use it for therapeutic purposes.

## Author Contributions

BL wrote the first draft. RH illustrated the draft. LZ modified the draft. The rest joined in the discussion.

## Conflict of Interest

The authors declare that the research was conducted in the absence of any commercial or financial relationships that could be construed as a potential conflict of interest.

## References

[B1] Aggarwal N. R. King L. S. D'Alessio F. R. (2014). Diverse macrophage populations mediate acute lung inflammation and resolution. Am. J. Physiol. Lung Cell Mol. Physiol. 306 (8), L709–L725. 10.1152/ajplung.00341.2013 24508730 PMC3989724

[B2] An X. Sun X. Yang X. Liu D. Hou Y. Chen H. (2019). Oxidative stress promotes ventilator-induced lung injury through activating NLRP3 inflammasome and TRPM2 channel. Artif. Cells Nanomed. Biotechnol. 47 (1), 3448–3455. 10.1080/21691401.2019.1652631 31411068

[B3] Aujla S. J. Chan Y. R. Zheng M. Fei M. Askew D. J. Pociask D. A. (2008). IL-22 mediates mucosal host defense against Gram-negative bacterial pneumonia. Nat. Med. 14 (3), 275–281. 10.1038/nm1710 18264110 PMC2901867

[B4] Baker P. J. Boucher D. Bierschenk D. Tebartz C. Whitney P. G. D'Silva D. B. (2015). NLRP3 inflammasome activation downstream of cytoplasmic LPS recognition by both caspase-4 and caspase-5. Eur. J. Immunol. 45 (10), 2918–2926. 10.1002/eji.201545655 26173988

[B5] Bergsbaken T. Fink S. L. Cookson B. T. (2009). Pyroptosis: host cell death and inflammation. Nat. Rev. Microbiol. 7 (2), 99–109. 10.1038/nrmicro2070 19148178 PMC2910423

[B6] Black R. A. Kronheim S. R. Merriam J. E. March C. J. Hopp T. P. (1989). A pre-aspartate-specific protease from human leukocytes that cleaves pro-interleukin-1 beta. J. Biol. Chem. 264 (10), 5323–5326. 10.1016/s0021-9258(18)83546-3 2784432

[B7] Burgos J. R. Iresjö B.-M. Olsson L. Smedh U. (2019). LPS immune challenge reduces arcuate nucleus TSHR and CART mRNA and elevates plasma CART peptides. BMC Neurosci. 20 (1), 59 10.1186/s12868-019-0539-z 31829131 PMC6907259

[B8] Butt Y. Kurdowska A. Allen T. C. (2016). Acute lung injury: a clinical and molecular review. Arch. Pathol. Lab Med. 140 (4), 345–350. 10.5858/arpa.2015-0519-RA 27028393

[B9] Campbell L. Raheem I. Malemud C. J. Askari A. D. (2016). The relationship between NALP3 and autoinflammatory syndromes. Int. J. Mol. Sci. 17 (5), 725 10.3390/ijms17050725 PMC4881547 27187378

[B10] Chen K. W. Groß C. J. Sotomayor F. V. Stacey K. J. Tschopp J. Sweet M. J. (2014). The neutrophil NLRC4 inflammasome selectively promotes IL-1β maturation without pyroptosis during acute Salmonella challenge. Cell Rep. 8 (2), 570–582. 10.1016/j.celrep.2014.06.028 25043180

[B11] Chen S. Wu Q. Zhong D. Li C. Du L. (2020). Caffeine prevents hyperoxia-induced lung injury in neonatal mice through NLRP3 inflammasome and NF-kappaB pathway. Respir. Res. 21 (1), 140 10.1186/s12931-020-01403-2 32513156 PMC7278162

[B12] Cheng K. T. Xiong S. Ye Z. Hong Z. Di A. Tsang K. M. (2017). Caspase-11-mediated endothelial pyroptosis underlies endotoxemia-induced lung injury. J. Clin. Invest. 127 (11), 4124–4135. 10.1172/JCI94495 28990935 PMC5663346

[B13] Cheng Z. Jiang X. Pansuria M. Fang P. Mai J. Mallilankaraman K. (2015). Hyperhomocysteinemia and hyperglycemia induce and potentiate endothelial dysfunction via μ-calpain activation. Diabetes 64 (3), 947–959. 10.2337/db14-0784 25352635 PMC4338586

[B14] Dalli J. Serhan C. N. (2012). Specific lipid mediator signatures of human phagocytes: microparticles stimulate macrophage efferocytosis and pro-resolving mediators. Blood 120 (15), e60–e72. 10.1182/blood-2012-04-423525 22904297 PMC3471524

[B15] Delaleu N. Bickel M. (2004). Interleukin-1 beta and interleukin-18: regulation and activity in local inflammation. Periodontology 35, 42–52. 10.1111/j.0906-6713.2004.003569.x 15107057

[B16] Distler J. H. W. Huber L. C. Gay S. Distler O. Pisetsky D. S. (2006). Microparticles as mediators of cellular cross-talk in inflammatory disease. Autoimmunity 39 (8), 683–690. 10.1080/08916930601061538 17178565

[B17] Duan R. Xie H. Liu Z. Z. (2020). The role of autophagy in osteoarthritis. Front Cell Dev. Biol. 8, 608388 10.3389/fcell.2020.608388 33324654 PMC7723985

[B18] Frank D. Vince J. E. (2019). Pyroptosis versus necroptosis: similarities, differences, and crosstalk. Cell Death Differ. 26 (1), 99–114. 10.1038/s41418-018-0212-6 30341423 PMC6294779

[B19] Fukumoto J. Fukumoto I. Parthasarathy P. T. Cox R. Huynh B. Ramanathan G. K. (2013). NLRP3 deletion protects from hyperoxia-induced acute lung injury. Am. J. Physiol. Cell Physiol. 305 (2), C182–C189. 10.1152/ajpcell.00086.2013 23636457 PMC3725631

[B20] Galam L. Rajan A. Failla A. Soundararajan R. Lockey R. F. Kolliputi N. (2016). Deletion of P2X7 attenuates hyperoxia-induced acute lung injury via inflammasome suppression. Am. J. Physiol. Lung Cell Mol. Physiol. 310 (6), L572–L581. 10.1152/ajplung.00417.2015 26747786 PMC4796258

[B21] Gao J. Peng S. Shan X. Deng G. Shen L. Sun J. (2019). Inhibition of AIM2 inflammasome-mediated pyroptosis by Andrographolide contributes to amelioration of radiation-induced lung inflammation and fibrosis. Cell Death Dis. 10 (12), 957 10.1038/s41419-019-2195-8 31862870 PMC6925222

[B22] Guo H. Callaway J. B. Ting J. P. Y. (2015). Inflammasomes: mechanism of action, role in disease, and therapeutics. Nat. Med. 21 (7), 677–687. 10.1038/nm.3893 26121197 PMC4519035

[B23] Harroch S. Gothelf Y. Revel M. Chebath J. (1995). 5' upstream sequences of MyD88, an IL-6 primary response gene in M1 cells: detection of functional IRF-1 and Stat factors binding sites. Nucleic Acids Res. 23 (17), 3539–3546. 10.1093/nar/23.17.3539 7567467 PMC307235

[B24] He X. Qian Y. Li Z. Fan E. K. Li Y. Wu L. (2016a). TLR4-Upregulated IL-1beta and IL-1RI promote alveolar macrophage pyroptosis and lung inflammation through an autocrine mechanism. Sci. Rep. 6, 31663 10.1038/srep31663 27526865 PMC4985817

[B25] He Y. Hara H. Nunez G. (2016b). Mechanism and regulation of NLRP3 inflammasome activation. Trends Biochem. Sci. 41 (12), 1012–1021. 10.1016/j.tibs.2016.09.002 27669650 PMC5123939

[B26] Hong C. Y. Zhang H. D. Liu X. Y. Xu Y. (2019). Attenuation of hyperoxic acute lung injury by Lycium barbarum polysaccharide via inhibiting NLRP3 inflammasome. Arch Pharm. Res. 42 (10), 902–908. 10.1007/s12272-019-01175-4 31388826

[B27] Hou L. Yang Z. Wang Z. Zhang X. Zhao Y. Yang H. (2018). NLRP3/ASC-mediated alveolar macrophage pyroptosis enhances HMGB1 secretion in acute lung injury induced by cardiopulmonary bypass. Lab. Invest. 98 (8), 1052–1064. 10.1038/s41374-018-0073-0 29884910

[B28] Huang L. S. Hong Z. Wu W. Xiong S. Zhong M. Gao X. (2020). mtDNA activates cGAS signaling and suppresses the YAP-mediated endothelial cell proliferation program to promote inflammatory injury. Immunity 52 (3), 475–486. 10.1016/j.immuni.2020.02.002 32164878 PMC7266657

[B29] Hung C.-M. Peng C.-K. Wu C.-P. Huang K.-L. (2018). Bumetanide attenuates acute lung injury by suppressing macrophage activation. Biochem. Pharmacol. 156, 60–67. 10.1016/j.bcp.2018.08.013 30102895

[B30] Irrera N. Russo M. Pallio G. Bitto A. Mannino F. Minutoli L. (2020). The role of NLRP3 inflammasome in the pathogenesis of traumatic brain injury. Int. J. Mol. Sci. 21 (17), 6204 10.3390/ijms21176204 PMC7503761 32867310

[B31] Jena K. K. Mehto S. Nath P. Chauhan N. R. Sahu R. Dhar K. (2020). Autoimmunity gene IRGM suppresses cGAS-STING and RIG-I-MAVS signaling to control interferon response. EMBO Rep. 21 (9), e50051 10.15252/embr.202050051 32715615 PMC7507369

[B32] Jin H.-Z. Yang X.-J. Zhao K.-L. Mei F.-C. Zhou Y. You Y.-D. (2019). Apocynin alleviates lung injury by suppressing NLRP3 inflammasome activation and NF-κB signaling in acute pancreatitis. Int. Immunopharm. 75, 105821 10.1016/j.intimp.2019.105821 31437787

[B33] Kao R. L. Xu X. Xenocostas A. Parry N. Mele T. Martin C. M. (2014). Induction of acute lung inflammation in mice with hemorrhagic shock and resuscitation: role of HMGB1. J. Inflamm. 11 (1), 30 10.1186/s12950-014-0030-7 PMC4193406 25309129

[B34] Kayagaki N. Warming S. Lamkanfi M. Vande Walle L. Louie S. Dong J. (2011). Non-canonical inflammasome activation targets caspase-11. Nature 479 (7371), 117–121. 10.1038/nature10558 22002608

[B35] Kayagaki N. Wong M. T. Stowe I. B. Ramani S. R. Gonzalez L. C. Akashi-Takamura S. (2013). Noncanonical inflammasome activation by intracellular LPS independent of TLR4. Science 341 (6151), 1246–1249. 10.1126/science.1240248 23887873

[B36] Kesavardhana S. Kanneganti T.-D. (2017). Mechanisms governing inflammasome activation, assembly and pyroptosis induction. Int. Immunol. 29 (5), 201–210. 10.1093/intimm/dxx018 28531279 PMC5890894

[B37] King A. R. Francis S. E. Bridgeman C. J. Bird H. Whyte M. K. Crossman D. C. (2003). A role for caspase-1 in serum withdrawal-induced apoptosis of endothelial cells. Lab. Invest. 83 (10), 1497–1508. 10.1097/01.lab.0000093096.62765.85 14563951

[B38] Kojima M. Gimenes-Junior J. A. Chan T. W. Eliceiri B. P. Baird A. Costantini T. W. (2018). Exosomes in postshock mesenteric lymph are key mediators of acute lung injury triggering the macrophage activation Toll-like receptor 4. Faseb. J. 32 (1). 10.1096/fj.201700488R 28855278

[B39] Kox M. Pompe J. C. Peters E. Vaneker M. van der Laak J. W. van der Hoeven J. G. (2011). α7 nicotinic acetylcholine receptor agonist GTS-21 attenuates ventilator-induced tumour necrosis factor-α production and lung injury. Br. J. Anaesth. 107 (4), 559–566. 10.1093/bja/aer202 21771746

[B40] Kuipers M. T. Aslami H. Janczy J. R. van der Sluijs K. F. Vlaar A. P. J. Wolthuis E. K. (2012). Ventilator-induced lung injury is mediated by the NLRP3 inflammasome. Anesthesiology 116 (5), 1104–1115. 10.1097/ALN.0b013e3182518bc0 22531249

[B41] LaCanna R. Liccardo D. Zhang P. Tragesser L. Wang Y. Cao T. (2019). Yap/Taz regulate alveolar regeneration and resolution of lung inflammation. J. Clin. Invest. 129 (5), 2107–2122. 10.1172/JCI125014 30985294 PMC6486331

[B42] Lai D. Tang J. Chen L. Fan E. K. Scott M. J. Li Y. (2018). Group 2 innate lymphoid cells protect lung endothelial cells from pyroptosis in sepsis. Cell Death Dis. 9 (3), 369 10.1038/s41419-018-0412-5 29511181 PMC5840374

[B43] Lamkanfi M. Dixit V. M. (2014). Mechanisms and functions of inflammasomes. Cell 157 (5), 1013–1022. 10.1016/j.cell.2014.04.007 24855941

[B44] Lamkanfi M. Sarkar A. Vande Walle L. Vitari A. C. Amer A. O. Wewers M. D. (2010). Inflammasome-dependent release of the alarmin HMGB1 in endotoxemia. J. immunol. 185 (7), 4385–4392. 10.4049/jimmunol.1000803 20802146 PMC3428148

[B45] Li D. Ren W. Jiang Z. Zhu L. (2018). Regulation of the NLRP3 inflammasome and macrophage pyroptosis by the p38 MAPK signaling pathway in a mouse model of acute lung injury. Mol. Med. Rep. 18 (5), 4399–4409. 10.3892/mmr.2018.9427 30152849 PMC6172370

[B46] Lin X. Y. Choi M. S. Porter A. G. (2000). Expression analysis of the human caspase-1 subfamily reveals specific regulation of the CASP5 gene by lipopolysaccharide and interferon-gamma. J. Biol. Chem. 275 (51), 39920–39926. 10.1074/jbc.M007255200 10986288

[B47] Liu H. Gu C. Liu M. Liu G. Wang D. Liu X. (2019). Ventilator-induced lung injury is alleviated by inhibiting NLRP3 inflammasome activation. Mol. Immunol. 111, 1–10. 10.1016/j.molimm.2019.03.011 30952009

[B48] Liu H. Yu X. Yu S. Kou J. (2015). Molecular mechanisms in lipopolysaccharide-induced pulmonary endothelial barrier dysfunction. Int. Immunopharm. 29 (2), 937–946. 10.1016/j.intimp.2015.10.010 26462590

[B49] López-Reyes A. Martinez-Armenta C. Espinosa-Velázquez R. Vázquez-Cárdenas P. Cruz-Ramos M. Palacios-Gonzalez B. (2020). NLRP3 inflammasome: the stormy link between obesity and COVID-19. Front. Immunol. 11, 570251 10.3389/fimmu.2020.570251 33193349 PMC7662564

[B50] Luo M. Hu L. Li D. Wang Y. He Y. Zhu L. (2017). MD-2 regulates LPS-induced NLRP3 inflammasome activation and IL-1beta secretion by a MyD88/NF-kappaB-dependent pathway in alveolar macrophages cell line. Mol. Immunol. 90, 1–10. 10.1016/j.molimm.2017.06.035 28654770

[B51] Malik A. Kanneganti T. D. (2017). Inflammasome activation and assembly at a glance. J. Cell Sci. 130 (23), 3955–3963. 10.1242/jcs.207365 29196474 PMC5769591

[B52] Man S. M. Karki R. Kanneganti T.-D. (2017a). Molecular mechanisms and functions of pyroptosis, inflammatory caspases and inflammasomes in infectious diseases. Immunol. Rev. 277 (1), 61–75. 10.1111/imr.12534 28462526 PMC5416822

[B53] Man S. M. Karki R. Kanneganti T. D. (2017b). Molecular mechanisms and functions of pyroptosis, inflammatory caspases and inflammasomes in infectious diseases. Immunol. Rev. 277 (1), 61–75. 10.1111/imr.12534 28462526 PMC5416822

[B54] Maniatis N. A. Orfanos S. E. (2008). The endothelium in acute lung injury/acute respiratory distress syndrome. Curr. Opin. Crit. Care 14 (1), 22–30. 10.1097/MCC.0b013e3282f269b9 18195622

[B55] Matikainen S. Nyman T. A. Cypryk W. (2020). Function and regulation of noncanonical caspase-4/5/11 inflammasome. J. Immunol. 204 (12), 3063–3069. 10.4049/jimmunol.2000373 32513874

[B56] Matthay M. A. Ware L. B. Zimmerman G. A. (2012). The acute respiratory distress syndrome. J. Clin. Invest. 122 (8), 2731–2740. 10.1172/JCI60331 22850883 PMC3408735

[B57] McIlwain D. R. Berger T. Mak T. W. (2013). Caspase functions in cell death and disease. Cold Spring Harb. Perspect. Biol. 5 (4), a008656 10.1101/cshperspect.a008656 23545416 PMC3683896

[B58] Miao E. A. Rajan J. V. Aderem A. (2011). Caspase-1-induced pyroptotic cell death. Immunol. Rev. 243 (1), 206–214. 10.1111/j.1600-065X.2011.01044.x 21884178 PMC3609431

[B59] Mitra S. Exline M. Habyarimana F. Gavrilin M. A. Baker P. J. Masters S. L. (2018). Microparticulate caspase 1 regulates gasdermin D and pulmonary vascular endothelial cell injury. Am. J. Respir. Cell Mol. Biol. 59 (1), 56–64. 10.1165/rcmb.2017-0393OC 29365280 PMC6039876

[B60] Mitra S. Wewers M. D. Sarkar A. (2015). Mononuclear phagocyte-derived microparticulate caspase-1 induces pulmonary vascular endothelial cell injury. PloS One 10 (12), e0145607 10.1371/journal.pone.0145607 26710067 PMC4692444

[B61] Mizushina Y. Shirasuna K. Usui F. Karasawa T. Kawashima A. Kimura H. (2015). NLRP3 protein deficiency exacerbates hyperoxia-induced lethality through Stat3 protein signaling independent of interleukin-1beta. J. Biol. Chem. 290 (8), 5065–5077. 10.1074/jbc.M114.603217 25548278 PMC4335242

[B62] Muendlein H. I. Jetton D. Connolly W. M. Eidell K. P. Magri Z. Smirnova I. (2020). cFLIP protects macrophages from LPS-induced pyroptosis via inhibition of complex II formation. Science 367 (6484), 1379–1384. 10.1126/science.aay3878 32193329 PMC7375259

[B63] Muller-Redetzky H. C. Suttorp N. Witzenrath M. (2014). Dynamics of pulmonary endothelial barrier function in acute inflammation: mechanisms and therapeutic perspectives. Cell Tissue Res. 355 (3), 657–673. 10.1007/s00441-014-1821-0 24599335 PMC7102256

[B64] Ning L. Wei W. Wenyang J. Rui X. Qing G. (2020). Cytosolic DNA-STING-NLRP3 axis is involved in murine acute lung injury induced by lipopolysaccharide. Clin. Transl. Med. 10 (7), e228 10.1002/ctm2.228 33252860 PMC7668192

[B65] Niu X. Zang L. Li W. Xiao X. Yu J. Yao Q. (2020). Anti-inflammatory effect of Yam Glycoprotein on lipopolysaccharide-induced acute lung injury via the NLRP3 and NF-κB/TLR4 signaling pathway. Int. Immunopharm. 81, 106024 10.1016/j.intimp.2019.106024 31784404

[B66] Oh C. Verma A. Aachoui Y. (2020). Caspase-11 non-canonical inflammasomes in the lung. Front. Immunol. 11, 1895 10.3389/fimmu.2020.01895 32973786 PMC7472987

[B67] Pandeya A. Li L. Li Z. Wei Y. (2019). Gasdermin D (GSDMD) as a new target for the treatment of infection. Med. Chem. Comm 10 (5), 660–667. 10.1039/c9md00059c PMC6533889 31191857

[B68] Peng W. Peng F. Lou Y. Li Y. Zhao N. Shao Q. (2020a). Autophagy alleviates mitochondrial DAMP-induced acute lung injury by inhibiting NLRP3 inflammasome. Life Sci. 265, 118833 10.1016/j.lfs.2020.118833 33275990

[B69] Peng Y. Wu Q. Tang H. Chen J. Wu Q. Yuan X. (2020b). NLRP3 regulated CXCL12 expression in acute neutrophilic lung injury. J. Inflamm. Res. 13, 377–386. 10.2147/JIR.S259633 32801831 PMC7399452

[B70] Qiu Z. He Y. Ming H. Lei S. Leng Y. Xia Z.-Y. (2019). Lipopolysaccharide (LPS) aggravates high glucose- and hypoxia/reoxygenation-induced injury through activating ROS-dependent NLRP3 inflammasome-mediated pyroptosis in H9C2 cardiomyocytes. J. Diab. Res. 2019, 8151836 10.1155/2019/8151836 PMC6398034 30911553

[B71] Qu L. Chen C. Chen Y. Li Y. Tang F. Huang H. (2019). High-mobility group box 1 (HMGB1) and autophagy in acute lung injury (ALI): a review. Med. Sci. Mon. Int. Med. J. Exp. Clin. Res. 25, 1828–1837. 10.12659/MSM.912867 PMC6423734 30853709

[B72] Ramirez M. L. G. Salvesen G. S. (2018). A primer on caspase mechanisms. Semin. Cell Dev. Biol. 82, 79–85. 10.1016/j.semcdb.2018.01.002 29329946 PMC6043420

[B73] Rogers C. Fernandes-Alnemri T. Mayes L. Alnemri D. Cingolani G. Alnemri E. S. (2017). Cleavage of DFNA5 by caspase-3 during apoptosis mediates progression to secondary necrotic/pyroptotic cell death. Nat. Commun. 8, 14128 10.1038/ncomms14128 28045099 PMC5216131

[B74] Rowe S. J. Allen L. Ridger V. C. Hellewell P. G. Whyte M. K. B. (2002). Caspase-1-deficient mice have delayed neutrophil apoptosis and a prolonged inflammatory response to lipopolysaccharide-induced acute lung injury. J. Immunol. 169 (11), 6401–6407. 10.4049/jimmunol.169.11.6401 12444148

[B75] Rubenfeld G. D. Caldwell E. Peabody E. Weaver J. Martin D. P. Neff M. (2005). Incidence and outcomes of acute lung injury. N. Engl. J. Med. 353 (16), 1685–1693. 10.1056/NEJMoa050333 16236739

[B76] Ryu J. C. Kim M. J. Kwon Y. Oh J. H. Yoon S. S. Shin S. J. (2017). Neutrophil pyroptosis mediates pathology of *P. aeruginosa* lung infection in the absence of the NADPH oxidase NOX2. Mucosal Immunol. 10 (3), 757–774. 10.1038/mi.2016.73 27554297

[B77] Samanta S. Zhou Z. Rajasingh S. Panda A. Sampath V. Rajasingh J. (2018). DNMT and HDAC inhibitors together abrogate endotoxemia mediated macrophage death by STAT3-JMJD3 signaling. Int. J. Biochem. Cell Biol. 102, 117–127. 10.1016/j.biocel.2018.07.002 30010012 PMC6309960

[B78] Sangiuliano B. Perez N. M. Moreira D. F. Belizario J. E. (2014). Cell death-associated molecular-pattern molecules: inflammatory signaling and control. Mediat. Inflamm. 2014, 821043 10.1155/2014/821043 PMC4130149 25140116

[B79] Sarkar A. Mitra S. Mehta S. Raices R. Wewers M. D. (2009). Monocyte derived microvesicles deliver a cell death message via encapsulated caspase-1. PloS One 4 (9), e7140 10.1371/journal.pone.0007140 19779610 PMC2744928

[B80] Shalini S. Dorstyn L. Dawar S. Kumar S. (2015). Old, new and emerging functions of caspases. Cell Death Differ. 22 (4), 526–539. 10.1038/cdd.2014.216 25526085 PMC4356345

[B81] Shao X.-F. Li B. Shen J. Wang Q.-F. Chen S.-S. Jiang X.-C. (2020). Ghrelin alleviates traumatic brain injury-induced acute lung injury through pyroptosis/NF-κB pathway. Int. Immunopharm. 79, 106175 10.1016/j.intimp.2019.106175 31918060

[B82] Shi J. Gao W. Shao F. (2017). Pyroptosis: gasdermin-mediated programmed necrotic cell death. Trends Biochem. Sci. 42 (4), 245–254. 10.1016/j.tibs.2016.10.004 27932073

[B83] Shojaie L. Iorga A. Dara L. (2020). Cell death in liver diseases: a review. Int. J. Mol. Sci. 21 (24), 9682 10.3390/ijms21249682 PMC7766597 33353156

[B84] Summers C. Singh N. R. White J. F. Mackenzie I. M. Johnston A. Solanki C. (2014). Pulmonary retention of primed neutrophils: a novel protective host response, which is impaired in the acute respiratory distress syndrome. Thorax 69 (7), 623–629. 10.1136/thoraxjnl-2013-204742 24706039 PMC4055272

[B85] Szekely B. Bossuyt V. Li X. Wali V. B. Patwardhan G. A. Frederick C. (2018). Immunological differences between primary and metastatic breast cancer. Ann. Oncol. 29 (11), 2232–2239. 10.1093/annonc/mdy399 30203045

[B86] Tan H. Jiang X. Yang F. Li Z. Liao D. Trial J. (2006). Hyperhomocysteinemia inhibits post-injury reendothelialization in mice. Cardiovasc. Res. 69 (1), 253–262. 10.1016/j.cardiores.2005.08.016 16226235 PMC4400842

[B87] Tang L. Zhang H. Wang C. Li H. Zhang Q. Bai J. (2017). M2A and M2C macrophage subsets ameliorate inflammation and fibroproliferation in acute lung injury through interleukin 10 pathway. Shock 48 (1), 119–129. 10.1097/SHK.0000000000000820 27941591

[B88] Tian X. Sun H. Casbon A. J. Lim E. Francis K. P. Hellman J. (2017). NLRP3 inflammasome mediates dormant neutrophil recruitment following sterile lung injury and protects against subsequent bacterial pneumonia in mice. Front. Immunol. 8, 1337 10.3389/fimmu.2017.01337 29163464 PMC5671513

[B89] Tulotta C. Stefanescu C. Chen Q. Torraca V. Meijer A. H. Snaar-Jagalska B. E. (2019). CXCR4 signaling regulates metastatic onset by controlling neutrophil motility and response to malignant cells. Sci. Rep. 9 (1), 2399 10.1038/s41598-019-38643-2 30787324 PMC6382824

[B90] Van Opdenbosch N. Gurung P. Vande Walle L. Fossoul A. Kanneganti T.-D. Lamkanfi M. (2014). Activation of the NLRP1b inflammasome independently of ASC-mediated caspase-1 autoproteolysis and speck formation. Nat. Commun. 5, 3209 10.1038/ncomms4209 24492532 PMC3926011

[B91] Van Opdenbosch N. Lamkanfi M. (2019). Caspases in cell death, inflammation, and disease. Immunity 50 (6), 1352–1364. 10.1016/j.immuni.2019.05.020 31216460 PMC6611727

[B92] Vassallo A. Wood A. J. Subburayalu J. Summers C. Chilvers E. R. (2019). The counter-intuitive role of the neutrophil in the acute respiratory distress syndrome. Br. Med. Bull. 131 (1), 43–55. 10.1093/bmb/ldz024 31504234

[B93] Wang S. Miura M. Jung Y. K. Zhu H. Li E. Yuan J. (1998). Murine caspase-11, an ICE-interacting protease, is essential for the activation of ICE. Cell 92 (4), 501–509. 10.1016/s0092-8674(00)80943-5 9491891

[B94] Wang Y. C. Liu Q. X. Zheng Q. Liu T. Xu X. E. Liu X. H. (2019). Dihydromyricetin alleviates sepsis-induced acute lung injury through inhibiting NLRP3 inflammasome-dependent pyroptosis in mice model. Inflammation 42 (4), 1301–1310. 10.1007/s10753-019-00990-7 30887396

[B95] Wang Y. Gao W. Shi X. Ding J. Liu W. He H. (2017a). Chemotherapy drugs induce pyroptosis through caspase-3 cleavage of a gasdermin. Nature 547 (7661), 99–103. 10.1038/nature22393 28459430

[B96] Wang Y. Liu X. Shi H. Yu Y. Yu Y. Li M. (2020). NLRP3 inflammasome, an immune-inflammatory target in pathogenesis and treatment of cardiovascular diseases. Clin. Transl. Med. 10 (1), e128 10.1002/ctm2.13 32508013 PMC7240865

[B97] Wang Y. Xu C. F. Liu Y. J. Mao Y. F. Lv Z. Li S. Y. (2017b). Salidroside attenuates ventilation induced lung injury via SIRT1-dependent inhibition of NLRP3 inflammasome. Cell. Physiol. Biochem. 42 (1), 34–43. 10.1159/000477112 28490015

[B98] Ware L. B. (2006). Pathophysiology of acute lung injury and the acute respiratory distress syndrome. Semin. Respir. Crit. Care Med. 27 (4), 337–349. 10.1055/s-2006-948288 16909368

[B99] Williams A. E. Chambers R. C. (2014). The mercurial nature of neutrophils: still an enigma in ARDS? Am. J. Physiol. Lung Cell Mol. Physiol. 306 (3), L217–L230. 10.1152/ajplung.00311.2013 24318116 PMC3920201

[B100] Winkler S. Rösen-Wolff A. (2015). Caspase-1: an integral regulator of innate immunity. Semin. Immunopathol. 37 (4), 419–427. 10.1007/s00281-015-0494-4 26059719

[B101] Wu D.-D. Pan P.-H. Liu B. Su X.-L. Zhang L.-M. Tan H.-Y. (2015). Inhibition of alveolar macrophage pyroptosis reduces lipopolysaccharide-induced acute lung injury in mice. Chin. Med. J. 128 (19), 2638–2645. 10.4103/0366-6999.166039 26415803 PMC4736856

[B102] Wu D. Pan P. Su X. Zhang L. Qin Q. Tan H. (2016). Interferon regulatory factor-1 mediates alveolar macrophage pyroptosis during LPS-induced acute lung injury in mice. Shock 46 (3), 329–338. 10.1097/SHK.0000000000000595 26939040 PMC4978602

[B103] Wu J. Yan Z. Schwartz D. E. Yu J. Malik A. B. Hu G. (2013). Activation of NLRP3 inflammasome in alveolar macrophages contributes to mechanical stretch-induced lung inflammation and injury. J. Immunol. 190 (7), 3590–3599. 10.4049/jimmunol.1200860 23436933 PMC3608749

[B104] Xi H. Zhang Y. Xu Y. Yang W. Y. Jiang X. Sha X. (2016). Caspase-1 inflammasome activation mediates homocysteine-induced pyrop-apoptosis in endothelial cells. Circ. Res. 118 (10), 1525–1539. 10.1161/circresaha.116.308501 27006445 PMC4867131

[B105] Xia S. (2020). Biological mechanisms and therapeutic relevance of the gasdermin family. Mol. Aspect. Med. 76, 100890 10.1016/j.mam.2020.100890 PMC7704569 32800355

[B106] Yang H. Lv H. Li H. Ci X. Peng L. (2019). Oridonin protects LPS-induced acute lung injury by modulating Nrf2-mediated oxidative stress and Nrf2-independent NLRP3 and NF-κB pathways. Cell Commun. Signal. 17 (1), 62 10.1186/s12964-019-0366-y 31186013 PMC6558832

[B107] Yang J. Zhao Y. Zhang P. Li Y. Yang Y. Yang Y. (2016). Hemorrhagic shock primes for lung vascular endothelial cell pyroptosis: role in pulmonary inflammation following LPS. Cell Death Dis. 7 (9), e2363 10.1038/cddis.2016.274 27607578 PMC5059873

[B108] Yang X. Sun X. Chen H. Xi G. Hou Y. Wu J. (2017). The protective effect of dopamine on ventilator-induced lung injury via the inhibition of NLRP3 inflammasome. Int. Immunopharm. 45, 68–73. 10.1016/j.intimp.2017.02.002 28189055

[B109] Yao L. Sun T. (2019). Glycyrrhizin administration ameliorates Streptococcus aureus-induced acute lung injury. Int. Immunopharm. 70, 504–511. 10.1016/j.intimp.2019.02.046 30884430

[B110] Yi Y.-S. (2017). Caspase-11 non-canonical inflammasome: a critical sensor of intracellular lipopolysaccharide in macrophage-mediated inflammatory responses. Immunology 152 (2), 207–217. 10.1111/imm.12787 28695629 PMC5588777

[B111] Zeng Y. Qin Q. Li K. Li H. Song C. Li Y. (2019). PKR suppress NLRP3-pyroptosis pathway in lipopolysaccharide-induced acute lung injury model of mice. Biochem. Biophys. Res. Commun. 519 (1), 8–14. 10.1016/j.bbrc.2019.08.054 31474337

[B112] Zhang L. Xu C. Chen X. Shi Q. Su W. Zhao H. (2018). SOCS-1 suppresses inflammation through inhibition of NALP3 inflammasome formation in smoke inhalation-induced acute lung injury. Inflammation 41 (4), 1557–1567. 10.1007/s10753-018-0802-y 29907905 PMC7102050

[B113] Zhang Y. Li X. Grailer J. J. Wang N. Wang M. Yao J. (2016). Melatonin alleviates acute lung injury through inhibiting the NLRP3 inflammasome. J. Pineal Res. 60 (4), 405–414. 10.1111/jpi.12322 26888116

[B114] Zhao L.-R. Xing R.-L. Wang P.-M. Zhang N.-S. Yin S.-J. Li X.-C. (2018). NLRP1 and NLRP3 inflammasomes mediate LPS/ATP-induced pyroptosis in knee osteoarthritis. Mol. Med. Rep. 17 (4), 5463–5469. 10.3892/mmr.2018.8520 29393464

[B115] Zhu W. London N. R. Gibson C. C. Davis C. T. Tong Z. Sorensen L. K. (2012). Interleukin receptor activates a MYD88-ARNO-ARF6 cascade to disrupt vascular stability. Nature 492 (7428), 252–255. 10.1038/nature11603 23143332 PMC3521847

